# Utility of CSF biomarkers in assessing neurodegeneration in Early‐Onset Alzheimer’s disease

**DOI:** 10.1002/alz.091628

**Published:** 2025-01-09

**Authors:** Mark C. Eldaief, Alexandra Touroutoglou, Michael Brickhouse, Yuta Katsumi, Ani Eloyan, Kelly N. Nudelman, Tatiana M. Foroud, Maria C. Carrillo, Gil D. Rabinovici, Liana G. Apostolova, Suzanne E. Schindler, Elizabeth M. Herries, Rachel L. Henson, Jeffrey L. Dage, Bradford C. Dickerson

**Affiliations:** ^1^ Massachusetts General Hospital and Harvard Medical School, Boston, MA USA; ^2^ Frontotemporal Disorders Unit and Massachusetts Alzheimer’s Disease Research Center, Department of Neurology, Massachusetts General Hospital and Harvard Medical School, Boston, MA USA; ^3^ Department of Biostatistics, Brown University, Providence, RI USA; ^4^ Department of Medical and Molecular Genetics, Indiana University School of Medicine, Indianapolis, IN USA; ^5^ Alzheimer's Association, Chicago, IL USA; ^6^ Department of Neurology, Memory and Aging Center, University of California San Francisco, San Francisco, CA USA; ^7^ Department of Radiology and Imaging Sciences, Indiana University School of Medicine, Indianapolis, IN USA; ^8^ Department of Neurology, Indiana University School of Medicine, Indianapolis, IN USA; ^9^ Department of Neurology, Washington University School of Medicine, St. Louis, MO USA; ^10^ Department of Neurology, Washington University School of Medicine, Saint Louis, MO USA; ^11^ Department of Neurology, Washington University School of Mediciine, Saint Louis, MO USA

## Abstract

**Background:**

There is a significant need for biomarkers of neurodegenerative burden in Early‐onset Alzheimer’s disease (EOAD). Evidence suggests that levels of specific CSF biomarkers (e.g., Neurofilament light (NfL), Synaptosomal‐Associated Protein (SNAP‐25), neurogranin, Visinin‐like protein 1 (VILIP‐1), Aß 42/40, phospho‐tau and total tau) index the extent of neurodegeneration in dementing illnesses. However, it remains unclear whether these biomarkers correlate to cortical atrophy patterns in EOAD. Based on prior work demonstrating correlations between NfL, SNAP‐25, neurogranin and Aß 42/40 CSF levels and cognitive impairment in EOAD (Dage et al. 2023), we hypothesized that these biomarkers (and not VILIP‐1, phospho‐tau or total tau) would variably predict cortical atrophy within our recently described EOAD signature (Touroutoglou et al. 2023).

**Method:**

We recruited 92 EOAD patients. In each patient, atrophy within the EOAD cortical signature were calculated as W‐scores (i.e., Z‐scores adjusted for age and sex relative to a sample of healthy controls). We first ran a simple regression analysis of each of the 7 CSF biomarkers and W‐scores in the EOAD signature across EOAD patients. We then entered the biomarkers with significant correlations to the EOAD signature into a stepwise regression analysis with backward elimination to ascertain the most parsimonious model predicting atrophy in the EOAD signature. As a control region not expected to be related to these biomarkers, we assessed correlations to calcarine fissure cortical atrophy.

**Result:**

As predicted, we observed a significant correlation between CSF levels of NfL, SNAP‐25, neurogranin and Aß 42/40 and atrophy within the EOAD signature. After entering these four biomarkers into the stepwise regression analysis, the most parsimonious model identified complementary contributions of NfL, SNAP‐25, and Aß 42/40, in predicting atrophy in the EOAD signature. There were no correlations between any biomarker and calcarine atrophy.

**Conclusion:**

Selected CSF biomarkers in EOAD patients predict the degree of atrophy within the EOAD cortical signature. Ongoing work includes correlating these biomarkers with topographical atrophy patterns on the whole‐brain voxel‐wise level. Our results suggest that certain CSF biomarkers could assess neurodegenerative burden within EOAD individuals. This would provide valuable information regarding disease progression for clinical care and clinical trials involving disease‐modifying therapies.

